# Identification of genes modified by N6-methyladenosine in patients with colorectal cancer recurrence

**DOI:** 10.3389/fgene.2022.1043297

**Published:** 2022-10-17

**Authors:** Qianru Zhu, Xingxing Huang, Shuxian Yu, Lan Shou, Ruonan Zhang, Han Xie, Zimao Liang, Xueni Sun, Jiao Feng, Ting Duan, Mingming Zhang, Yu Xiang, Xinbing Sui, Weiwei Jin, Lili Yu, Qibiao Wu

**Affiliations:** ^1^ State Key Laboratory of Quality Research in Chinese Medicines, Faculty of Chinese Medicine, Macau University of Science and Technology, Macau, China; ^2^ School of Pharmacy, Hangzhou Normal University, Hangzhou, Zhejiang, China; ^3^ Guangdong-Hong Kong-Macao Joint Laboratory for Contaminants Exposure and Health, Guangdong University of Technology, Guangzhou, Guangdong, China; ^4^ Key Laboratory of Elemene Class Anti-Cancer Chinese Medicines, Engineering Laboratory of Development and Application of Traditional Chinese Medicines, Collaborative Innovation Center of Traditional Chinese Medicines of Zhejiang Province, Hangzhou Normal University, Hangzhou, Zhejiang, China; ^5^ Zhuhai MUST Science and Technology Research Institute, Zhuhai, Guangdong, China; ^6^ Department of Gastrointestinal-Pancreatic Surgery, Zhejiang Provincial People’s Hospital, People’s Hospital of Hangzhou Medical College, Hangzhou, Zhejiang, China; ^7^ Key Laboratory of Gastroenterology of Zhejiang Province, Hangzhou, Zhejiang, China

**Keywords:** colorectal cancer, m^6^A methylation modification, tumor immune microenvironment, recurrence, overall survival

## Abstract

**Background:** Recent studies demonstrate that N6-methyladenosine (m^6^A) methylation plays a crucial role in colorectal cancer (CRC). Therefore, we conducted a comprehensive analysis to assess the m^6^A modification patterns and identify m^6^A-modified genes in patients with CRC recurrence.

**Methods:** The m^6^A modification patterns were comprehensively evaluated by the NMF algorithm based on the levels of 27 m^6^A regulators, and tumor microenvironment (TME) cell-infiltrating characteristics of these modification patterns were systematically assessed by ssGSEA and CIBERSORT algorithms. The principal component analysis algorithm based on the m^6^A scoring scheme was used to explore the m^6^A modification patterns of individual tumors with immune responses. The weighted correlation network analysis and univariable and multivariable Cox regression analyses were applied to identify m^6^A-modified gene signatures. The single-cell expression dataset of CRC samples was used to explore the tumor microenvironment affected by these signatures.

**Results:** Three distinct m^6^A modification patterns with significant recurrence-free survival (RFS) were identified in 804 CRC patients. The TME characterization revealed that the m^6^A modification pattern with longer RFS exhibited robust immune responses. CRC patients were divided into high- and low-score subgroups according to the m^6^A score individually, which was obtained from the m^6^A-related signature genes. The patients with low m^6^A scores had both longer RFS and overall survival (OS) with altered immune cell infiltration. Notably, m^6^A-modified genes showed significant differences related to the prognosis of CRC patients in the meta-GEO cohort and TCGA cohort. Single-cell expression indicated that ALVRL1 was centrally distributed in endothelial tip cells and stromal cells.

**Conclusion:** The m^6^A modification plays an indispensable role in the formation of TME diversity and complexity. Importantly, the signatures (TOP2A, LRRC58, HAUS6, SMC4, ACVRL1, and KPNB1) were identified as m^6^A-modified genes associated with CRC recurrence, thereby serving as a promising predictive biomarker or therapeutic target for patients with CRC recurrence.

## Introduction

Colorectal cancer (CRC) is the most common gastrointestinal malignancy and remains the main cause of cancer-related death worldwide (Siegel et al., 2021). Currently, the 5-year survival rate of CRC patients has been improved along with the development of new chemotherapeutics and advanced techniques. However, high recurrence and unsatisfactory prognosis are still major problems of CRC, due to delayed diagnosis and adverse drug effects (Sanoff et al., 2008; Hashiguchi et al., 2020). Currently, it has been recognized that CRC with recurrence is associated with genetic, genomic, and epigenetic changes (Lao and Grady, 2011). Therefore, identifying the crucial tumor biomarkers to predict the prognosis of CRC is urgently required.

N6-methyladenosine (m^6^A), as a reversible epigenetic reprogramming, is extensively modified in a variety of RNAs, comprising mRNAs, tRNAs, and snRNAs, as well as long-chain non-coding RNAs (Dominissini et al., 2012; Liang et al., 2020). m^6^A modification on RNA is abundant near the stop codon and 3-untranslated region (3-UTR) (Meyer et al., 2012; Ke et al., 2015) and translated near the 5-UTR in a cap-independent manner (Meyer et al., 2015), thereby regulating RNA transcription, translation, and metabolism. m^6^A modifications occur *via* signal transduction enzyme, methyltransferase, and demethylase, which are regarded as the “reader,” “writer,” and “eraser,” respectively. Specifically, “writers” can install the methyl to target RNAs, which include METTL3 (Schumann et al., 2016), METTL5 (Huang et al., 2022), METTL14 (Liu et al., 2014), WTAP (Ping et al., 2014), and RBM15/15B (Meyer and Jaffrey, 2017), and “erasers” mainly include FTO (Jia et al., 2011) and ALKBH5 (Zheng et al., 2013), which both selectively remove the methyl from certain RNAs. “Readers” such as YTHDC1, YTHDC2 (Haussmann et al., 2016), YTHDF1, YTHDF2, YTHDF3 (Huang et al., 2022), EIF3A (Meyer and Jaffrey, 2017), IGF2BP1, IGF2BP2, IGF2BP3 (Huang et al., 2018), HNRNPC (Huang et al., 2021), HNRNPA2B1 (Jiang et al., 2021), G3BP1, G3BP2 (Arguello et al., 2017), ELAVL1 (Pan et al., 2021), PRRC2A (Wu et al., 2019), and FMR1 (Worpenberg et al., 2021) can decipher the m^6^A methylation codes.

An increasing number of studies have revealed the relationship among the m^6^A modification, tumor microenvironment (TME) (Li et al., 2021), and chemotherapy resistance (Gu et al., 2021). Depletion of YTHDF1 in dendritic cells significantly enhances antigen presentation, resulting in CD8+T cell activation (Han et al., 2019). Macrophage-specific METTL14 knockout drives CD8+T cell differentiation in a dysfunctional direction, impairing the ability of CD8+T cells to eliminate tumors (Dong et al., 2021). METTL3 mediated gemcitabine, 5-fluorouracil, and cisplatin resistance in non-small-cell lung cancer and pancreatic cancer (Taketo et al., 2018; Jin et al., 2019). In addition, the m^6^A regulator-based methylation modification pattern led to different TME immune profiles in colorectal cancer (Chong et al., 2021). In addition, the relationship between m^6^A modification and TME characteristics and clinical prognosis in primary glioblastomas was elucidated completely by Cai et al. (2021). Therefore, m^6^A mRNA regulators and distinct modification patterns play an important role in the development or function of immune cells, which also promote resistance to chemotherapy and recurrence of the tumor. However, the relationship between m^6^A regulation and the recurrence status of CRC remains largely unknown.

In this study, we performed a comprehensive analysis of the expression of 27 m^6^A RNA methylation regulators using integrated data from the GEO database of patients with CRC. Afterward, consensus clustering analysis based on the gene expression of 27 m^6^A RNA methylation regulators was utilized to distinguish three different m^6^A modification subgroups. Then, the relationship between m^6^A modification patterns and immune infiltration related to CRC recurrence was systematically assessed. Furthermore, m^6^A regulators related to targeted mRNAs were screened by WGCNA analysis and regression analysis, and we identified a promising m^6^A-modified prognostic signature that can effectively predict the clinical outcomes of patients with CRC. Hence, our study suggests that the m^6^A methylation modification pattern contributes to the tumor immune microenvironment and has clinical prognostic value for colorectal cancer patients, providing novel insights into the diagnosis and treatment of CRC.

## Materials and methods

### Collection of publicly attainable expression datasets

The gene expression matrix and clinical traits of colorectal cancer patients were downloaded from the Gene Expression Omnibus database (https://www.ncbi.nlm.nih.gov/geo/), and a total of 804 patients were enrolled for analysis, including those from the GSE39582 (*N* = 558) (Marisa et al., 2013), GSE72970 (*N* = 95) (Del Rio et al., 2017), and GSE103479 (*N* = 151) (Allen et al., 2018). The ComBat method from the “SVA” R package was widely used to remove the batch effects between the different GEO datasets (Dai et al., 2018) to form meta-GEO datasets. Copy number variations of the TCGA-COAD were obtained from the UCSC Xena database (https://gdc-hub.s3.us-east-1.amazonaws.com/download/TCGA-COAD.cnv.tsv.gz). The package “Rcircos” was employed in R studio to plot the copy number variation landscape of 27 m^6^A regulators in human chromosomes. The clinical information and m^6^A regulator expression of the meta-GEO and the TCGA data are listed in Supplementary Tables S1, S2.

### Non-negative matrix factorization clustering

Next, to assess the differences of m^6^A regulation between the different CRC clusters, the meta-GEO cohort was used for non-negative matrix factorization clustering, a method that can classify samples better than consensus clustering. Also then, the NMF rank survey and consensus heatmap were used to evaluate the optimal k value, and the meta-GEO cohort was divided into three clusters. Kaplan–Meier survival analysis was used to evaluate recurrence-free and overall survival between the different clusters.

### Gene set variation analysis and GO analysis

We utilized GSVA analysis to identify underlying signaling pathways that are differentially stimulated behind the different m^6^A modification patterns in the meta-GEO cohort (Hänzelmann et al., 2013). The well-defined molecular biological signatures (h.all.v2022.1. Hs.symbols.gmt) were derived from the Hallmarker gene set (http://www.gsea/msigdb.org/gsea/msigdb) (Subramanian et al., 2005). In addition, Gene Ontology, a functional enrichment analysis, including three components: cellular components, biological processes, and molecular functions, was performed for understanding the underlying biological meaning of key genes extracted by WGCNA.

### Estimation of immune cell infiltration by the ssGSEA and deconvolution algorithm

Initially, we used single-sample gene set enrichment analysis (ssGSEA) to quantify the relative abundance of 23 immune cell types behind the tumor microenvironment among the different m^6^A-modified clusters. Special feature gene panels for marking each immune cell type were curated from a recent study (Charoentong et al., 2017; Jia et al., 2018). The relative abundance of each immune cell type is represented by an enrichment fraction in the ssGSEA analysis and normalized to a uniform distribution. Afterward, CIBERSORT (Newman et al., 2015) (http://cibersort.stanford.edu/), a deconvolution approach, was used to estimate the abundances of 22 distinct leukocyte fractions with the gene expression profile of colorectal cancer patients individually, and participants with *p* value <0.05 were considered for differential analysis of leukocyte fraction between the m^6^A low-risk group and the m^6^A high-risk group.

### Extraction of mRNAs from transcription profiles

Three microarray datasets from different platforms were matched with each GPL annotation file to explore m^6^A-targeted mRNAs. The probes for GSE39582 and GSE72970 are extracted from the Affymetrix HG-U133_Plus 2.0 microarray, and for GSE103479, the probes are extracted from the Almac Diagnostics Custom Xcel array. Subsequently, we extracted probe sets annotated with “protein coding” in the GENCODE project by matching the GENCODE (release 39). Finally, a total of 16,346 mRNAs in the meta-GEO cohorts were obtained for subsequent analysis.

### Weighted correlation network analysis

First, Pearson’s correlation analysis between 27 m^6^A regulators and m^6^A-targeted mRNAs was performed, and 6,771 m^6^A-targeted mRNAs were identified (cor > 0.3, *p* < 0.05). Then, weighted correlation network analysis (WGCNA) was performed to acquire the required gene modules based on the gene expressions and patient traits by using the R “WGCNA” package (Langfelder and Horvath, 2008). First, a soft thresholding power value was calculated to produce a scale-free network topology. Hereafter, one-step network construction and detection of consensus modules were executed. Also, the similarity modules were assigned. Finally, correlations between clinical traits (age, sex, recurrence events, recurrence-free survival, status, and OS) and each module were calculated.

### Identification of the m^6^A-modified gene with prognostic value

Univariable and multivariable Cox regression analyses were utilized to narrow the gene range and maximize the accuracy (Huang et al., 2018), Subsequently, we selected the meaning genes from the multivariate Cox regression analysis and analyzed the overall survival and recurrence-free survival probability of these genes in the meta-GEO and the TCGA cohort.

### Single-cell RNA-seq analysis

To explore the tumor microenvironment affected by m^6^A-modified signatures, we downloaded the cell plots of single-cell sequencing of colorectal tumor samples and adjacent non-tumor samples from Single-Cell Expression Altas ((https://www.ebi.ac.uk/gxa/sc/home), and the parameters of drawing path are as follows: plot type: UMAP, ploy options: n_neibors:100, color plot by: ontology labels, gene names: by ALVRL1 and HAUS6.

### Statistical analyses

Statistical analyses in this study were performed using R-4.1.2. Student's t-test or Wilcoxon rank-sum test was used to estimate the quantitative data for normally distributed or non-normally distributed data, respectively. The Kruskal–Wallis test and one-way analysis of variance were used for the comparison of the three distinct groups for the non-parametric and parametric data, respectively. The association between the m^6^A cluster and prognosis, risk group, and prognosis was analyzed by Kaplan–Meier survival analysis and the Cox proportional hazard model with the R package “Survminer” (0.4.9). The survival-cutoff function from the “survival” package in R studio was applied to stratify CRC patients into low-risk and high-risk subgroups. The alpha level for all comparisons was 0.05, and the Benjamini–Hochberg method was applied to control for the false discovery rate for multiple hypothesis testing procedures (Hazra and Gogtay, 2016).

## Results

### The landscape of expression variation of m^6^A regulators in colorectal cancer recurrence patients

**FIGURE 1:**
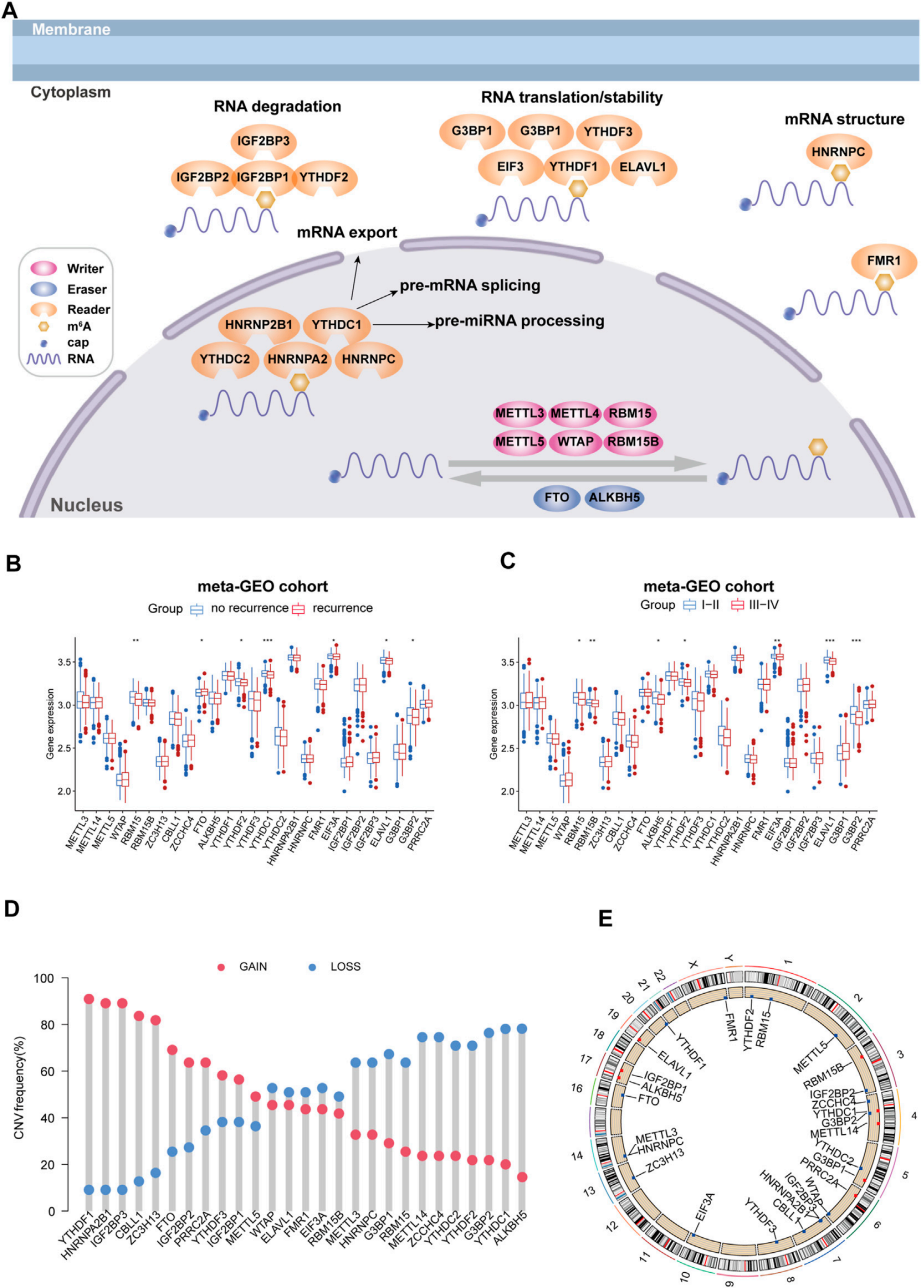
Landscape of genetic and expression variation of m^6^A regulators in colorectal cancer recurrence population. **(A)** Regulation of m^6^A regulation and its biological functions in RNA metabolism. **(B)** Expression change of m^6^A regulators in colorectal cancer with recurrence compared with no recurrence. **(C)** Expression changes of m^6^A regulators in colorectal cancer with high stage compared with low stage. *p < 0.05, **p < 0.01, and ***p < 0.001. **(D)** CNV variation frequency of m^6^A regulators in the TCGA cohort. The height of the column represented the alteration frequency. The deletion frequency is represented by a blue dot; The amplification frequency is represented by a red dot. **(E)** Location of CNV alteration of m^6^A regulators on 23 chromosomes using the TCGA cohort.

In this study, we investigated the differential expression of 27 m^6^A RNA methylation regulatory genes, including “writes,” “readers,” and “erasers” (Figure 1A), between the recurrence group and no recurrence group, low-stage group (stage I/II), and high-stage group (stage III/IV) of colorectal cancer tissue by using a dataset from the meta-GEO cohort. We found that some m^6^A RNA methylation regulators were significantly linked to the recurrence and stage status in patients with CRC (Figures 1B,C). Then, in the TCGA COAD cohort, we performed the copy number variation (CNV) analysis alterations of m^6^A regulators in CRC patients with recurrence. For CNV events, approximately 59% (16/27) of m^6^A regulators lost DNA copy number, with YTHDC1 having the highest degree of copy number loss. Eleven m^6^A regulators gained copy number, among which YTHDF1 had the highest percentage increase (Figure 1D). The m^6^A regulator CNV alterations and locations on chromosomes are shown in Figure 1E.

### Determining the relationship between m^6^A-modified patterns and prognosis of CRC patients

**FIGURE 2:**
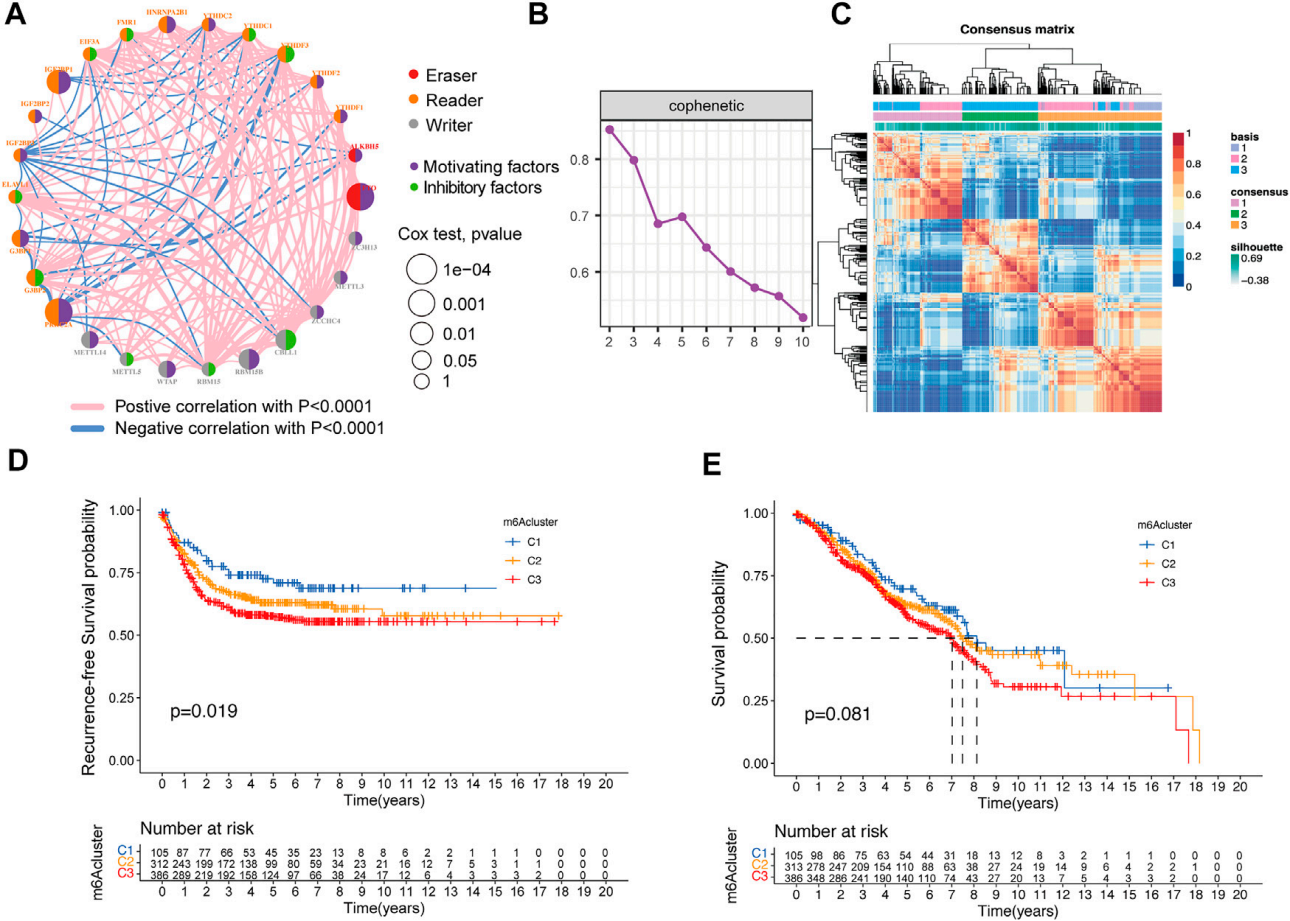
Relationship between the m^6^A methylation modification pattern and prognostic characteristics. **(A)** Interaction of expression on 27 m^6^A regulators in colorectal cancer. The m^6^A regulators in three RNA modification clusters were depicted by circles in different colors. Readers, orange; writers, gray; erasers, red. The lines connecting m^6^A regulators represented their interaction with each other. The size of each circle represented the recurrence effect of each regulator and was scaled by the *p*-value. Inhibitory factors for patients’ recurrence were indicated by a green right semicircle and motivating factors indicated by a purple right semicircle. **(B)** NMF rank survey result. **(C)** NMF analysis identification of the three m^6^A modification clusters. **(D,E)** Kaplan–Meier curves of recurrence-free survival **(D)** and overall survival **(E)** for 804 CRC patients in the meta-GEO cohort with different m^6^A cluster patterns. The numbers of patients in m^6^A-cluster 1, m^6^A-cluster 2, and m^6^A-cluster 3 three phenotypes are 105, 313, and 386, respectively.

Three GEO datasets (GSE39582, GSE72970, and GSE103479) with gene expression and available relapse-free survival time, overall survival time, and clinical traits were enrolled in our meta-GEO cohort. The comprehensive network of interactions of the 27 m^6^A regulators and the recurrence status of CRC patients is shown in Figure 2A. The results firmly indicate that these regulators played a critical role in the recurrence of CRC. Then, the NMF algorithm was used to divide 804 patients into different m^6^A clusters, according to the expression of 27 m^6^A regulators. Then, we adopted three clusters as an acceptable criterion according to the results of the NMF rank survey (Figures 2B,C), and then, the meta-GEO cohort was divided into three distinct clusters according to the expression of 27 m^6^A regulators by using the NMF algorithm, including 105 cases in “cluster 1,” 313 cases in “cluster 2,” and 386 cases in “cluster 3” (Supplementary Table S2). Importantly, the Kaplan–Meier survival analysis revealed that cluster 1 had better recurrence-free probability (*p* = 0.019) than cluster 2 and cluster 3 (Figure 2D). However, cluster 1 also showed longer overall survival than the other two clusters, but there was no significant statistical difference (*p* = 0.081) (Figure 2E).

### Immune profiles among the distinct m^6^A methylation-modified patterns

**FIGURE 3:**
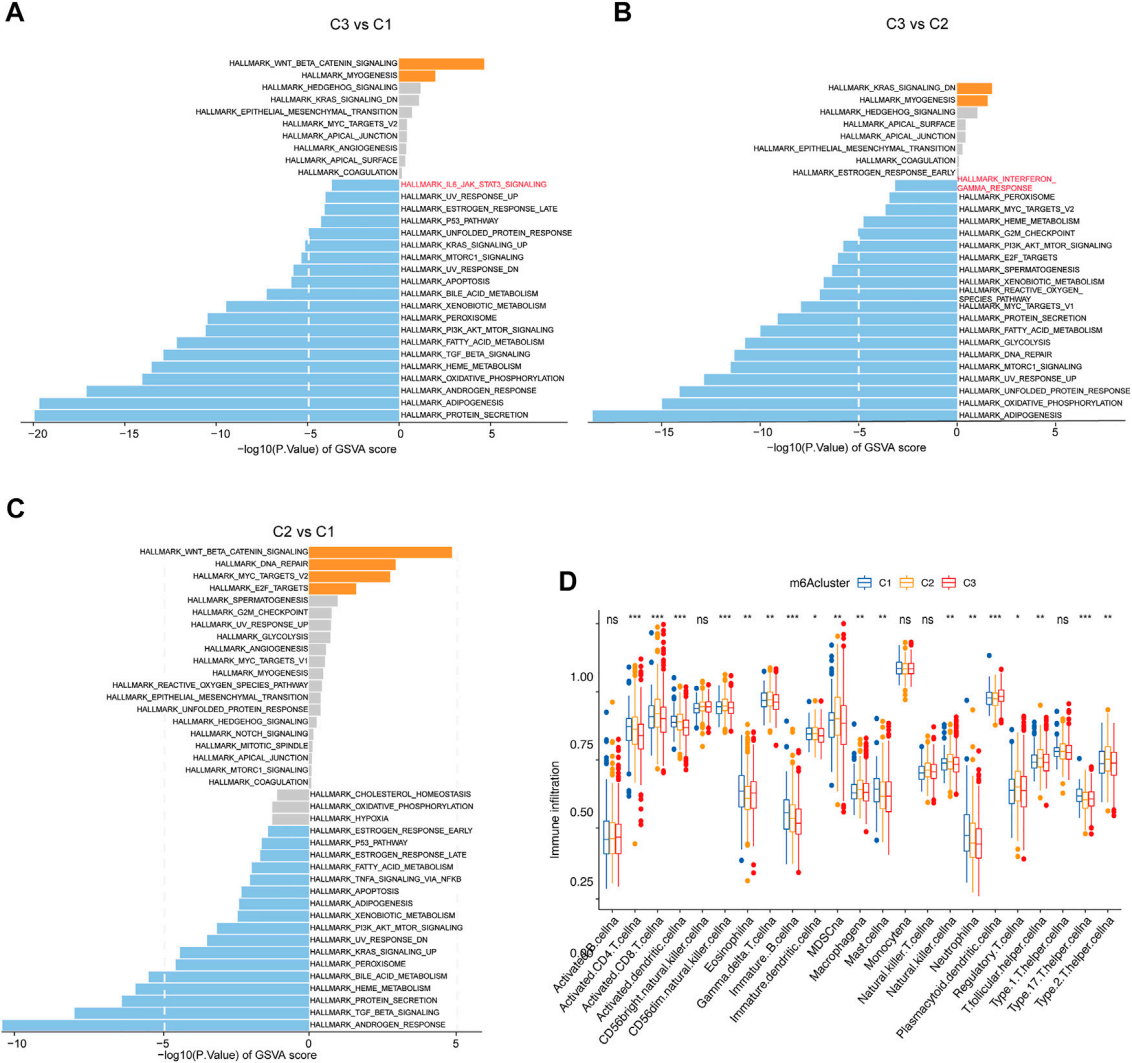
Immune profiles among the different m6A methylation modification patterns. **(A–C)** GSVA enrichment analysis shows the activation states of biological pathways in the three clusters. The biological processes are visualized with the bar plot: orange represents activated pathways; blue represents inhibited pathways. **(D)** Fraction of tumor-infiltrating lymphocyte cells in three m6A clusters using the ssGSEA algorithm. Within each group, the scattered dots represented TME cell expression values. The thick line represented the median value. The bottom and top of the boxes were the 25th and 75th percentiles, respectively (interquartile range). The statistical difference between the three gene clusters was compared through the Kruskal–Wallis H test. *p < 0.05; **p < 0.01; ***p < 0.001.

The aforementioned findings confirmed that the different clusters were significantly associated with the outcomes of CRC patients. Then, GSVA enrichment analysis was performed to explore the underlying molecular mechanisms behind three different clusters. Intriguingly, we found that cluster 2, compared with cluster 1, was highly enriched in beta-catenin signaling, DNA repair, MYC target, and E2F targets pathway, whereas it was downregulated in androgen response and TGF beta signaling transduction. In addition, cluster 3 was significantly upregulated in beta-catenin signaling and the myogenesis-related pathway. Remarkably, cluster 3 showed that the IL6 JAK STAT3 signaling pathway was activated when compared to cluster 1 and downregulated in interferon-gamma response when compared to cluster 2 (Figures 3A–C). Furthermore, to better-understand the association between immune profiles and m^6^A modification, we compared and visualized the relative abundances of 23 immune infiltrating cell subpopulations among three m^6^A modification patterns by using the ssGSEA algorithm. By contrast, cluster 1 was markedly enriched in innate and adaptive immune cell infiltration, including activated CD4 T cell, activated CD8 T cell, activated dendritic cell, CD56 dim natural killer cell, type 17 helper cell, and gamma delta T cell (Figure 3D).

### Construction of the m^6^A score and exploration of its clinical relevance

**FIGURE 4:**
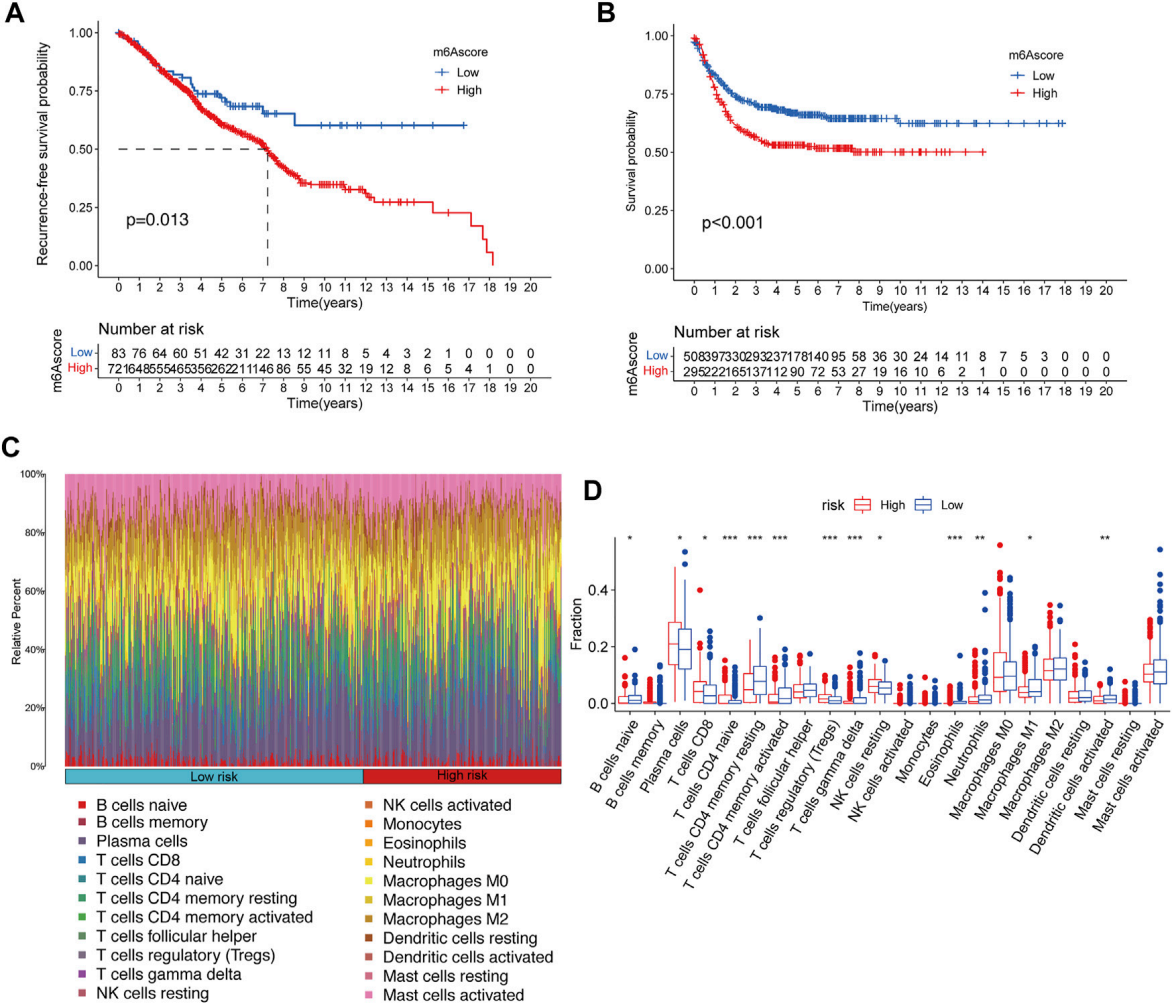
Construction of m^6^A signatures and TME cell infiltration analysis. **(A,B)** Kaplan–Meier curves of recurrence-free survival **(A)** and overall survival **(B)** for 804 CRC patients in the meta-GEO cohort with different m^6^A scores. **(C)** Bar plot visualizes the relative percent of 22 immune cells in each sample. **(D)** Boxplot of all 22 immune cells differentially infiltrated fraction. *p < 0.05; **p < 0.01; ***p < 0.001.

For the assessed m^6^A score of each CRC patient, a total of 195 differential expression genes were extracted among the distinct m^6^A clusters, and then univariable Cox regression analysis was performed to screen key genes, which were related to the recurrence-free survival (Supplementary Table S3). Subsequently, the m^6^A score of each patient was calculated according to the PCA algorithm (Supplementary Table S2). The KM survival plot was performed to evaluate the relationship between the low-/high-m^6^A score group and prognosis. Importantly, patients in the low-m^6^A score group exhibited significantly longer recurrence-free time and survival time than those in the high-m^6^A score group (Figures 4A,B).

### TME immune cell infiltration characteristics in distinct m^6^A risk groups

Furthermore, to better-understand the underlying immune regulatory mechanisms of the different m^6^A score groups, hierarchical clustering was performed to explore the distinct patterns of tumor immune cell infiltration based on the immune cell fractions of CRC samples with CIBERSORT *p* < 0.05 between the high-m^6^A risk group and the low-m^6^A score group, and the tumor immune cell proportions by two clusters are shown in Figure 4C. We compared the fractions of 22 immunocytes between the high-m^6^A score group and low-m^6^A score group, and 13 immunocytes were altered in the low-m^6^A score group, including eight increasing immunocyte fractions (B cell naïve, T cells CD4 memory resting, T cells CD4 memory activated, T cells gamma–delta, eosinophils, neutrophils, M1 macrophage, and dendritic cells activated) and five decreasing immunocyte fractions (plasma cell, T cell CD8, T cell CD4 naïve, T cells regulatory, and NK cell resting) (Figure 4D).

### Functional enrichment and WGCNA analysis in m^6^A-related genes

**FIGURE 5:**
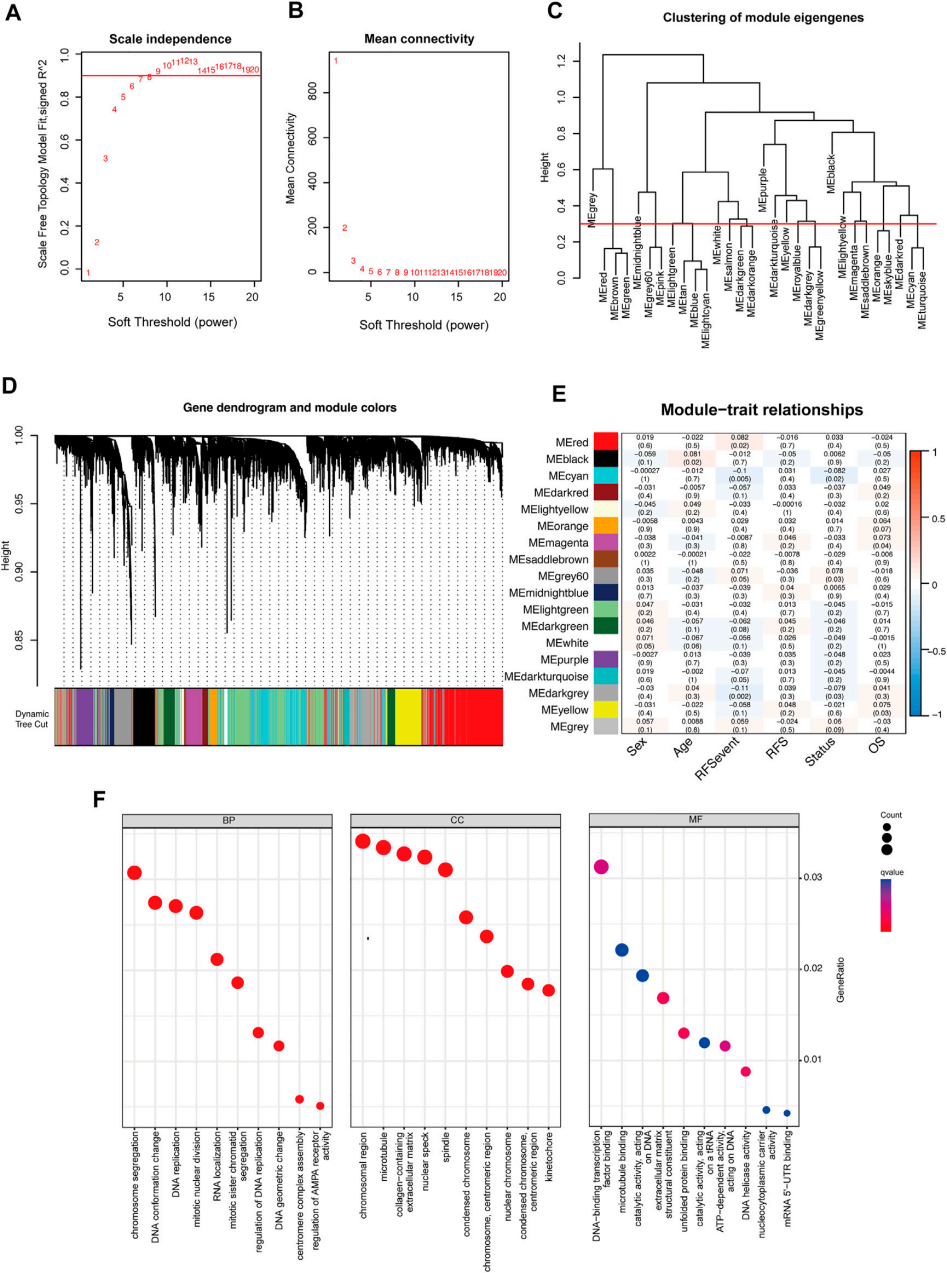
Weighted gene correlation network analysis of m^6^A methylation regulators. **(A,B)** Analysis of network topology to determine soft-thresholding power. **(C)** Eigengene dendrogram identified groups of correlated modules. **(D)** Gene dendrogram was obtained by clustering the dissimilarity based on consensus topological overlap with the corresponding module colors indicated by the color row. Each colored row represents a color-coded module that contains a group of highly connected genes. **(E)** Heatmap of the correlation between the module eigengenes and clinical traits of colorectal cancer. We selected the module red, cyan, gray60, dark turquoise, and dark gray blocks for subsequent analysis. **(F)** Gene Ontology analysis of genes in module red, cyan, gray60, dark turquoise, and dark gray.

A total of 6,770 m^6^A modified genes were obtained by Pearson’s correlation analysis (cor > 0.3, *p* < 0.05) (Supplementary Table S4). Then, these genes were used for weighted gene co-expression network analysis, which is commonly used to analyze the relationship between co-expression modules and external sample traits (Hazra and Gogtay, 2016). In our research, value “8” was selected as a soft thresholding power value because it produced a high similarity with a scale-free network and contributed to gene clustering (Figures 5A,B). Then, a one-step network to estimate the relationship between modules and clinical characteristics (sex, age, RFS events, RFS, status, and OS) was constructed. Here, a clustering dendrogram of m^6^A-related genes (Figures 5C,D) and 18 modules were obtained. Remarkably, module red, cyan, gray60, dark turquoise, and dark gray were significantly correlated to the recurrence-free survival and positively linked to the recurrence event in colorectal cancer, and these modules were adopted for further regression analysis (Figure 5E)**.** These significant modules were shown upregulated in chromosome segregation, RNA localization, and mRNA 5′-UTR binding by using Gene Ontology analysis (Figure 5F).

### Identification of m^6^A- and recurrence-related genes

**FIGURE 6:**
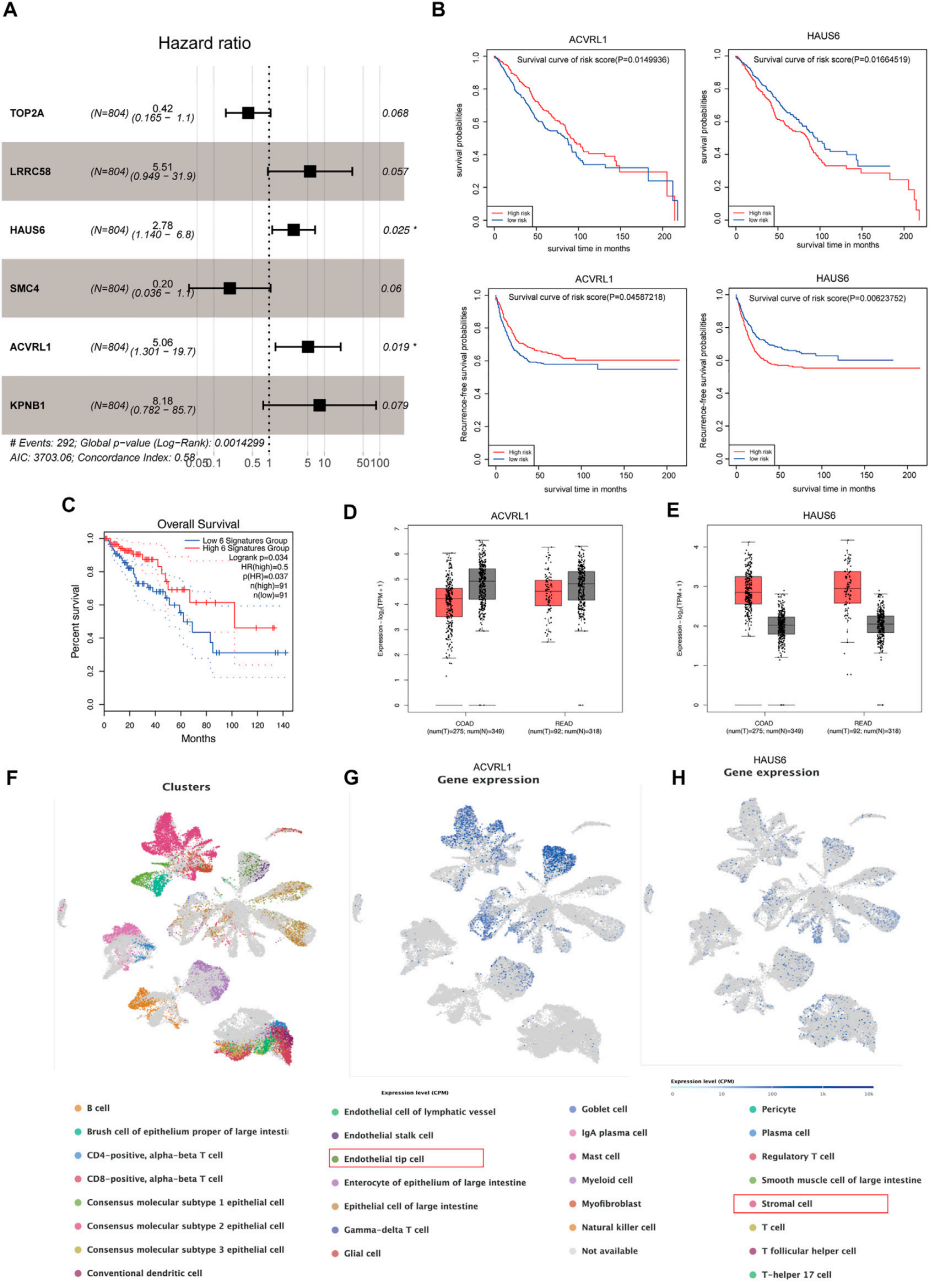
Identification of key genes modified by m6A. **(A)** Multivariable Cox regression analyses in the meta-GEO cohort by using the RFS model. **(B)** Survival plot of the significant genes obtained by multivariable Cox regression, including ACVRL1 and HAUS6. **(C)** Overall survival of the signature was obtained by multivariable Cox regression from the meta-GEO cohort in the TCGA cohort. **(D,E)** Gene expression of ACVRL1 and HAUS6 in the TCGA cohort. **(F)** Thirty Q21 clusters of the single-cell RNA-seq analysis. **(G,H)** Distribution of ACVRL1 and HAUS6 in colorectal cancer patients.

Initially, m^6^A-modified and recurrence-related genes were screened by WGCNA, then the univariable Cox analysis was carried out to attain the genes that were significantly correlated to recurrence and overall survival, and we acquired 225 genes (Supplementary Table S4). Subsequently, the multivariable Cox regression was adopted to determine the final prognostic factors by using 37 significant genes from the univariable Cox regression (*p* < 0.01) (Figure 6A), and six genes were recognized linked to CRC patient prognosis, namely, TOP2A, LRRC58, HAUS6, SMC4, ACVRL1, and KPNB1. We also found that ALVRL1 and HAUS6 were significantly related to the prognosis of CRC patients, including recurrence-free survival and overall survival (Figure 6B). Next, to evaluate the accuracy of the signatures obtained by the multivariable Cox regression analysis from the meta-GEO cohort, we downloaded the overall survival of these genes of CRC patients in the TCGA cohort from the GEPIA database. Importantly, these signatures were also significantly associated with the survival of the CRC patients (*p* = 0.034) (Figure 6C), and we also found that ALVRL1 and HAUS6 were differentially expressed in COAD and READ patients when compared to those in the normal sample in the TCGA cohort (Figures 6D,E). Finally, to confirm the underlying tumor microenvironment affected by ALVRL1 and HAUS6, the transcriptomes of single cells from CRC samples were downloaded from Single-Cell Expression Altas (https://www.ebi.ac.uk/gxa/sc/experiments/E-MTAB-8410/results/tsne), and the results indicated that ALVRL1 was centrally distributed in endothelial tip cells and stromal cells, whereas HAUS6 did not show central distribution (Figures 6F,G,H).

## Discussion

Based on accumulating evidence, dysregulation of m^6^A RNA methylation regulators, especially the m^6^A modification pattern, assumed an indispensable role in the occurrence and recurrence of the tumor. As most studies focus on a single regulator, the integrated patterns to decipher the characteristics of m^6^A regulators and underlying TME infiltration are not fully recognized in CRC patients with recurrence. Therefore, we explored the relationship between the distinct m^6^A modification patterns and CRC recurrence and built an m^6^A-based risk model to predict the prognosis of CRC.

In the current study, we analyzed and compared the expression of 27 key m^6^A RNA methylation regulators in CRC tissues with recurrence and no recurrence, and we observed differential expression levels of m^6^A regulators (RBM15, FTO, YTHDF2, YTHDC1, EIF3A, ELAVL1, and G3BP2) both in the recurrence tissues and in the high-stage tissues, indicating their potential functions as tumor motivators in CRC tumorigenesis and recurrence. Next, considering the universality and importance of the m^6^A modification pattern, we performed consensus clustering of 27 m^6^A RNA regulators and identified three subgroups: m^6^A cluster 1, m^6^A cluster 2, and m^6^A cluster 3. m^6^A cluster subgroups were verified to influence recurrence-free survival and overall survival. The immune profile analysis underlying distinct m^6^A clusters revealed that immune responses, including innate immunity and adaptive immunity, were enhanced in m^6^A cluster 1 with longer recurrence-free survival, and tumor immune cells were also enriched in m^6^A cluster 1.

To quantitatively illustrate the m^6^A signature, we calculated the m^6^A score of CRC patients individually by PCA based on 32 significantly prognostic m^6^A phenotype-related DEGs between the three m^6^A cluster subgroups. Also, the lower m^6^A score was significantly associated with better prognosis both in RFS and OS. Furthermore, the m^6^A score was negatively correlated with five of 23 immune-associated cells. CIBERSORT results also showed that B cell naïve, T cells CD4 memory resting, T cells CD4 memory activated, T cells gamma–delta, eosinophils, neutrophils, and dendritic cells activated elevated significantly in the low-risk group (lower m^6^A score group), which indicates a potential mechanism by which the m^6^A signature protects against CRC progression is by positively regulating immune cell infiltration.

Subsequently, WGCNA analysis, indeed, verified the connection between CRC recurrence and m^6^A regulator-related genes. Hence, we identified the prognostic value of m^6^A-modified gene signatures (TOP2A, LRRC58, HAUS6, SMC4, ACVRL1, and KPNB1) which were selected by the univariable and multivariable Cox regression analyses. Notably, these signatures also showed a significant relation to the survival in the TCGA cohort. In addition, the significant genes (ACVRL1 and HAUS6) obtained from multivariable Cox regression could serve as biomarkers for CRC patient survival. The single-cell expression dataset (Lee et al., 2020) of colorectal cancer samples demonstrated that ALVRL1 was centrally distributed in endothelial tip cells and stromal cells, whereas HAUS6 did not show central distribution, which also indicates that m^6^A target mRNAs affected the tumor microenvironment, thereby influencing the prognosis of CRC.

## Conclusion

We evaluated the m^6^A modification patterns of 804 primary CRC patients based on 27 m^6^A regulators and revealed the biological mechanism behind the distinct m^6^A modification patterns, which will help improve our understanding of the characteristics of TME cell infiltration and predict clinical prognosis. The m^6^A modification plays an indispensable role in the formation of TME diversity and complexity. Notably, TOP2A, LRRC58, HAUS6, SMC4, ACVRL1, and KPNB1 were identified as m^6^A-modified genes associated with CRC recurrence, thereby serving as a promising predictive biomarker panel or therapeutic target for patients with CRC recurrence.

## Data Availability

The original contributions presented in the study are included in the article/Supplementary Material; further inquiries can be directed to the corresponding authors.
